# Identification of iron metabolism-related genes in coronary heart disease and construction of a diagnostic model

**DOI:** 10.3389/fcvm.2024.1409605

**Published:** 2024-11-14

**Authors:** Lin Zhu, Jianxin Zhang, Wenhui Fan, Chen Su, Zhi Jin

**Affiliations:** Department of Traditional Chinese Medicine, The Second Hospital of Shandong University, Jinan, China

**Keywords:** coronary heart disease, iron metabolism, diagnostic model, immune infiltration, WGCNA

## Abstract

**Background:**

Coronary heart disease is a common cardiovascular disease, yferroptosiset its relationship with iron metabolism remains unclear.

**Methods:**

Gene expression data from peripheral blood samples of patients with coronary heart disease and a healthy control group were utilized for a comprehensive analysis that included differential expression analysis, weighted gene co-expression network analysis, gene enrichment analysis, and the development of a logistic regression model to investigate the associations and differences between the groups. Additionally, the CIBERSORT algorithm was employed to examine the composition of immune cell types within the samples.

**Results:**

Eight central genes were identified as being both differentially expressed and related to iron metabolism. These central genes are mainly involved in the cellular stress response. A logistic regression model based on the central genes achieved an AUC of 0.64–0.65 in the diagnosis of coronary heart disease. A higher proportion of M0 macrophages was found in patients with coronary heart disease, while a higher proportion of CD8T cells was observed in the normal control group.

**Conclusion:**

The study identified important genes related to iron metabolism in the pathogenesis of coronary heart disease and constructed a robust diagnostic model. The results suggest that iron metabolism and immune cells may play a significant role in the development of coronary heart disease, providing a basis for further research.

## 1 Introduction

Coronary heart disease (CAD) is a cardiovascular disease, often caused by arteriosclerosis. Arteriosclerosis is characterized by the deposition of cholesterol and calcium in the inner layer of the arterial walls, forming plaques that narrow the arterial lumen and obstruct blood flow (1). Coronary heart disease primarily affects the coronary arteries, which are responsible for supplying oxygen and nutrients to the heart (2). When the coronary arteries are damaged, the heart muscle may not receive enough oxygen and nutrients, leading to myocardial ischemia and angina. In severe cases, coronary heart disease can lead to myocardial infarction, where heart muscle tissue dies due to lack of blood supply (3). Symptoms of coronary heart disease include chest pain, shortness of breath, palpitations, and fatigue. Coronary heart disease is a common and deadly disease, significantly affecting patients' quality of life and lifespan.

In recent years, an increasing number of studies have focused on the important role of iron metabolism in the development and progression of CAD (4). As an essential trace element in the human body, iron plays a crucial role in various physiological processes, including oxygen transport, energy metabolism, and cell signaling (5). However, imbalances in iron metabolism, whether iron overload or deficiency, can have significant adverse effects on the cardiovascular system (5). Iron is a highly redox-active metal that can participate in the Fenton reaction, producing large amounts of hydroxyl radicals (·OH). These highly reactive free radicals can attack cell membranes, proteins, and DNA, leading to widespread tissue damage (5). In blood vessels, this oxidative damage directly compromises the integrity of endothelial cells, increases vascular permeability, and promotes the infiltration of inflammatory cells, thereby accelerating the process of atherosclerosis. Excess iron ions can catalyze the oxidation of low-density lipoprotein (LDL) (6). Oxidized LDL is more easily phagocytosed by macrophages than normal LDL, leading to the formation of foam cells. These foam cells are major components of atherosclerotic plaques, and their accumulation can cause narrowing of the vascular lumen, reduced blood flow, and increased risk of myocardial ischemia (7). Excess iron can stimulate the production and release of inflammatory factors such as interleukin-6 (IL-6), tumor necrosis factor-α (TNF-α), and C-reactive protein (CRP). These inflammatory factors not only directly participate in the formation and destabilization of atherosclerotic plaques but can also further activate immune cells, creating a vicious cycle that continuously exacerbates the vascular inflammatory state (8, 9). Healthy vascular endothelium is crucial for maintaining vascular tone and preventing thrombosis. Excess iron can directly damage endothelial cells and reduce the bioavailability of nitric oxide (NO). NO is an important vasodilator, and its reduction can lead to increased vascular contractility, decreased blood flow, and consequently increased risk of myocardial ischemia (10, 11). Ferroptosis is a newly discovered iron-dependent form of programmed cell death characterized by excessive intracellular iron accumulation and lipid peroxidation (12). In CAD, especially in myocardial ischemia-reperfusion injury, ferroptosis may be an important mechanism of myocardial cell damage. These mechanisms work together to potentially accelerate the formation of atherosclerosis, increase plaque instability, and ultimately lead to cardiovascular events (4, 13). On the other hand, iron deficiency can also increase the risk of CAD. The most direct consequence of iron deficiency is anemia, which leads to reduced oxygen-carrying capacity of the blood (14). In patients with already narrowed coronary arteries, anemia can further exacerbate myocardial hypoxia, increasing the frequency and severity of angina attacks. Long-term anemia can also lead to compensatory cardiac enlargement, increasing cardiac load. Iron is an important component of many iron-containing enzymes that play key roles in mitochondrial respiratory chain and ATP generation processes (15, 16). Iron deficiency may affect the energy metabolism of myocardial cells, reducing cardiac contractility and overall function. Studies have shown that iron deficiency may increase platelet activation and aggregation. Activated platelets are more prone to form thrombi, which is particularly dangerous in coronary arteries with existing atherosclerosis and may lead to acute coronary syndrome (16, 17).

It is worth noting that the relationship between iron metabolism and CAD presents a “U-shaped” curve, meaning that both high and low iron levels may be detrimental to cardiovascular health. This complex relationship provides new insights into the prevention and treatment of CAD while also presenting challenges. A deeper understanding of the interaction mechanisms between iron metabolism and CAD not only helps to elucidate the pathogenesis of the disease but may also provide important bases for developing new diagnostic markers and treatment strategies.

**Figure 1:**
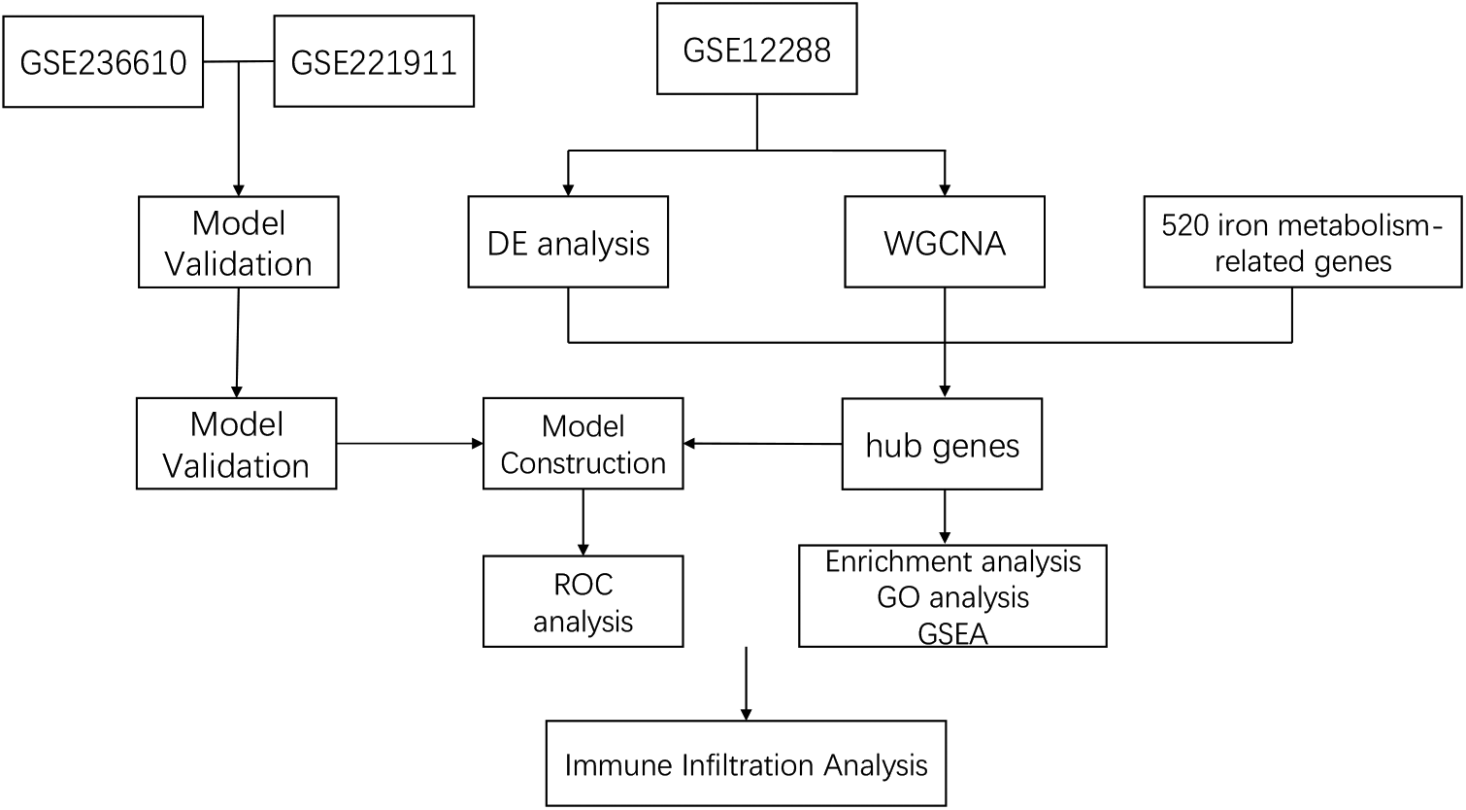
Study flowchart.

Leveraging abundant public datasets and bioinformatics approaches, this research pinpointed hub genes associated with iron metabolism and coronary artery disease (CAD) through differential analysis and weighted gene co-expression network analysis (WGCNA). It proceeded to develop a diagnostic model for CAD using stepwise and logistic regression analyses, which was subsequently validated across two additional blood sample datasets. The model demonstrated robust diagnostic capabilities, underscoring its potential utility in the clinical identification of CAD. Additionally, the study delved into immune infiltration within these samples, examining the relationship between various immune factors and the identified hub genes, our study design process is illustrated in Figure 1. This investigation lays the groundwork for understanding the significance of iron metabolism in the context of CAD, offering a novel perspective for future research.

## 2 Methods and materials

### 2.1 Data source

The gene expression data related to coronary heart disease was sourced from the publicly accessible NCBI Gene Expression Omnibus (GEO) database, under the specific study dataset identifier GSE12288 (18). This dataset examined the gene expression patterns in the peripheral blood of 110 individuals with coronary heart disease and 112 healthy controls. Additional validation was conducted using data from GSE236610 (19) and GSE221911 (20). Data related to ferroptosis were obtained from the public FerrDb database (http://www.zhounan.org/ferrdb), including genes that promote, inhibit, or are markers of ferroptosis, with a total of 264 unique genes after removing duplicates (21). In our sample selection process, we exclusively included studies focusing on CHD to minimize interference from other conditions. We prioritized studies with larger sample sizes to obtain more comprehensive data. Regarding demographic matching, we selected studies that demonstrated balanced control of age range, gender ratio, and ethnic composition, while also recording and matching lifestyle factors. In terms of comorbidities, we specifically chose studies that included participants with relevant comorbid conditions (such as hypertension, diabetes, and dyslipidemia). This careful selection strategy was implemented to ensure the robustness and reliability of our findings. By focusing on CHD-specific studies with well-documented comorbidities, we aimed to better understand the disease's characteristics while acknowledging the common coexistence of these conditions in real-world clinical settings. The inclusion of larger sample sizes enhanced the statistical power of our analysis, while the balanced demographic matching helped minimize potential confounding factors. This methodological approach allowed us to generate more reliable and clinically relevant results while maintaining the ecological validity of our research.

### 2.2 Differential expression analysis

The analysis of differential gene expression was conducted utilizing the “limma” package (version 3.48.3) in R (version 4.1.2), comparing samples from the group with coronary heart disease to those from the control group. The raw gene expression data were first normalized using the quantile normalization method. Genes were identified as differentially expressed (DEGs) based on adjusted *p*-values (*p*_adj) < 0.05, calculated using the Benjamini-Hochberg and Bonferroni method to control for false discovery rate, and an absolute log2 fold change (|log2FC|) greater than 0.585 (corresponding to a linear fold change of 1.5). Visual representations of these DEGs were generated using the “pheatmap” package (version 1.0.12) for heatmaps and the “ggplot2” package (version 3.3.5) for volcano plots. The heatmap utilized hierarchical clustering with complete linkage method and Euclidean distance metric, displaying the top 100 DEGs ranked by adjusted *p*-value. The volcano plot showed log2 fold change on the *x*-axis and -log10(adjusted *p*-value) on the *y*-axis, with upregulated genes colored red, downregulated genes colored blue, and non-significant genes colored gray. Gene names for the top 50 most significantly differentially expressed genes were labeled on the plot. All analyses were performed on a high-performance computing cluster with 64 GB RAM and 16 CPU cores to ensure efficient processing of large-scale gene expression data.

### 2.3 Weighted gene co-expression network analysis (WGCNA)

The “WGCNA” package (version 1.70-3) was utilized in R (version 4.1.2) to construct gene co-expression networks and pinpoint differentially expressed genes. As a cornerstone in systems biology, WGCNA (Weighted Gene Co-expression Network Analysis) detects and categorizes gene co-expression patterns, grouping genes into modules based on expression similarities. In this study, we employed a soft-thresholding power of 6, determined by the scale-free topology criterion (*R*^2^ > 0.8). The minimum module size was set to 30 genes, and the deep split parameter was set to 2 for medium sensitivity in cluster splitting. Modules were identified using the dynamic tree-cutting algorithm with a height cutoff of 0.25. The module eigengene dissimilarity threshold for merging was set at 0.25. Pearson correlation was used to calculate the adjacency matrix, and the topological overlap matrix (TOM) was computed using the TOMsimilarity function. Gene significance was calculated as the absolute correlation between gene expression and trait data, while module membership was determined by correlating gene expression profiles with module eigengenes. The network was shown using Cytoscape (version 3.8.2), with a edge weight cutoff of 0.2 to display only strong connections. This process enhances the analysis of gene expression data, facilitating the discovery of gene correlations and clusters linked to various biological states, ultimately providing insights into the underlying molecular mechanisms of coronary heart disease (22).

### 2.4 Gene enrichment analysis

This study pinpointed crucial genes linked to ferroptosis and coronary heart disease by leveraging the “VennDiagram” package in R to identify commonalities among Differentially Expressed Genes (DEGs), genes found via Weighted Gene Co-expression Network Analysis (WGCNA), and genes involved in iron metabolism. The expression differences of these essential genes between the coronary heart disease group and control subjects were illustrated using violin plots, with the significance of these variations determined through *t*-tests or Mann–Whitney *U* tests (*p* < 0.05). Furthermore, an enrichment analysis of these pivotal genes was carried out to explore their association with coronary heart disease. Gene Ontology (GO) analysis was performed to uncover their roles in biological processes (BP), with the results shown through the “GOplot” package in R.

### 2.5 Logistic regression model

In this study, logistic regression, a common method in automated disease diagnosis, was leveraged to distinguish between samples with coronary heart disease and control samples, marked by response variables 1 and 0, respectively. The analysis commenced with a stepwise regression to refine the model, focusing on eliminating non-significant predictors while keeping those of importance. This method enhances the model by iteratively adding or removing variables based on the optimization of the Akaike Information Criterion (AIC), ensuring a streamlined and effective predictor set. Following this, logistic regression was applied to understand the significant predictors' relationship with the occurrence of coronary heart disease. The model's diagnostic accuracy was evaluated by generating a receiver operating characteristic (ROC) curve and computing the area under this curve (AUC), utilizing R's “stats” and “pROC” packages for this purpose.

### 2.6 Immune infiltration analysis

The CIBERSORT algorithm was applied to evaluate the infiltration of immune cells within the microenvironment. This analysis encompassed a comprehensive set of 547 biomarkers and included 22 distinct human immune cell types, ranging from plasma cells, B cells, and T cells to various subgroups of bone marrow cells. The methodology of this computational tool involves conducting deconvolution analysis on the expression matrix of immune cells, leveraging the principles of linear support vector regression for accurate assessment and characterization of immune cell populations.

### 2.7 Statistical analysis

All statistical analyses were performed using R software (version 4.1.2) for comprehensive data processing and analysis. The selection of statistical tests strictly adhered to data distribution characteristics: initially, the Shapiro-Wilk test and *Q*–*Q* plots were employed to assess data normality. For continuous variables following normal distribution (such as certain gene expression data), independent samples *t*-tests were conducted for between-group comparisons. For non-normally distributed data (such as some immune cell infiltration indices), non-parametric Mann–Whitney *U* tests (also known as Wilcoxon rank-sum tests) were applied for statistical inference.

To control the false discovery rate (FDR) in multiple comparisons, both Benjamini-Hochberg and Bonferroni methods were implemented for *p*-value adjustment. For differential expression analysis, the “limma” package (version 3.48.3) was utilized for data processing, and quantile normalization was applied to standardize the raw gene expression data, effectively removing batch effects and systematic biases.

In the Weighted Gene Co-expression Network Analysis (WGCNA), Pearson correlation coefficients were calculated to evaluate gene-gene expression correlations, enabling the identification of highly interconnected gene modules. For immune cell infiltration analysis, the CIBERSORT algorithm was employed to assess the distribution of 22 immune cell types, followed by appropriate statistical methods to compare differences between groups.

This comprehensive statistical framework ensured robust and reliable results while maintaining appropriate control over type I error rates across multiple statistical comparisons. All statistical tests were two-sided, with statistical significance defined as *p* < 0.05, unless otherwise specified.

## 3 Results

### 3.1 Acquisition of iron metabolism central genes related to coronary heart disease

**Figure 2:**
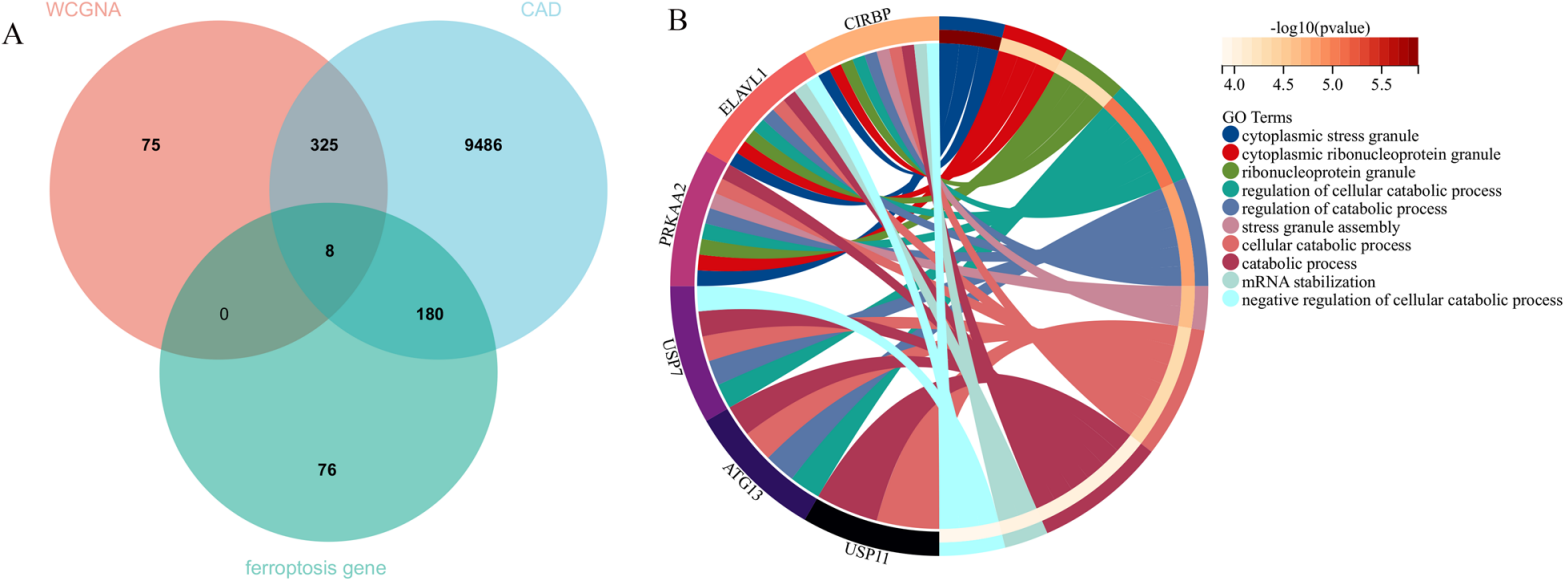
Gene expression differences between CAD and control samples. **(A)** Genes with significantly higher expression in CAD are marked in red, those with significantly higher expression in the control group are in blue, and genes with no significant changes are in gray. **(B)** Displays the top 50 genes with significantly higher expression in either CAD or control group samples.

**Figure 3:**
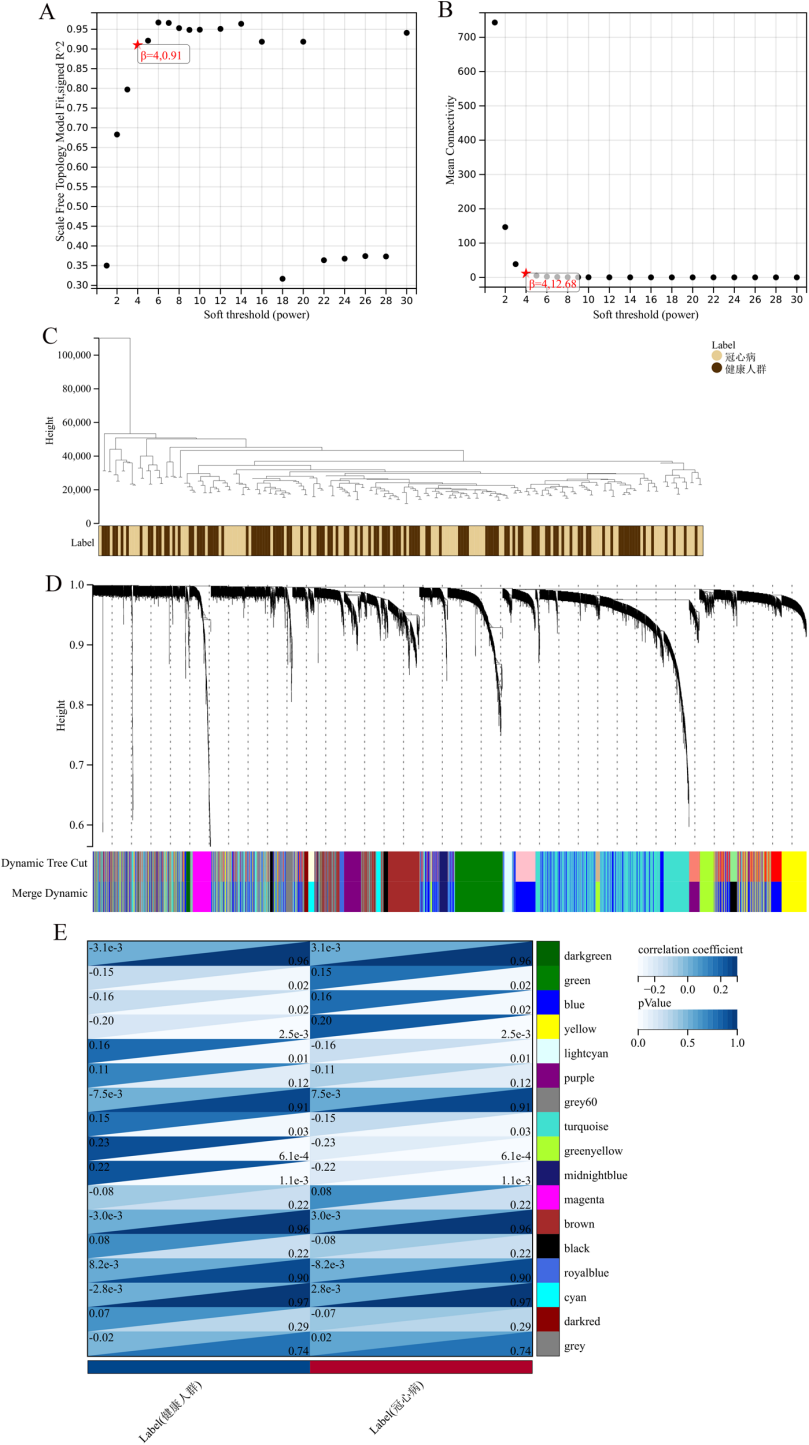
WGCNA analysis results. **(A)** Displays the scale-free fit index for various soft-thresholding powers. **(B)** Shows the average connectivity for different soft-thresholding powers. **(C)** A dendrogram of sample clustering. **(D)** A dendrogram showing gene clustering. **(E)** The correlation between different gene modules and clinical features.

The application of the criteria “*p*_adj < 0.05, |logFC| > 0.585” resulted in the identification of 9,487 differentially expressed genes from the GSE12288 dataset. The top 50 differentially expressed genes were shown in a volcano plot (Figure 2A) and heatmap (Figure 2B). After removing outliers and filtering genes, a weighted gene co-expression network was constructed using a dataset comprising 12,347 genes and 211 samples. Setting the soft-thresholding power to 4 led to a scale independence of 0.91 (Figure 3A) and an average connectivity of 12.68 (Figure 3B).

**Figure 4:**
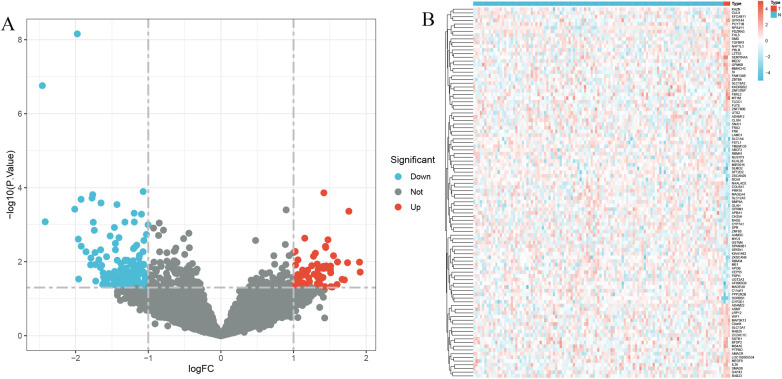
Central genes and functional analysis. **(A)** Identification of eight central genes through the intersection of DEGs, WGCNA, and genes related to iron metabolism. **(B)** The biological processes involving these central genes.

**Figure 5:**
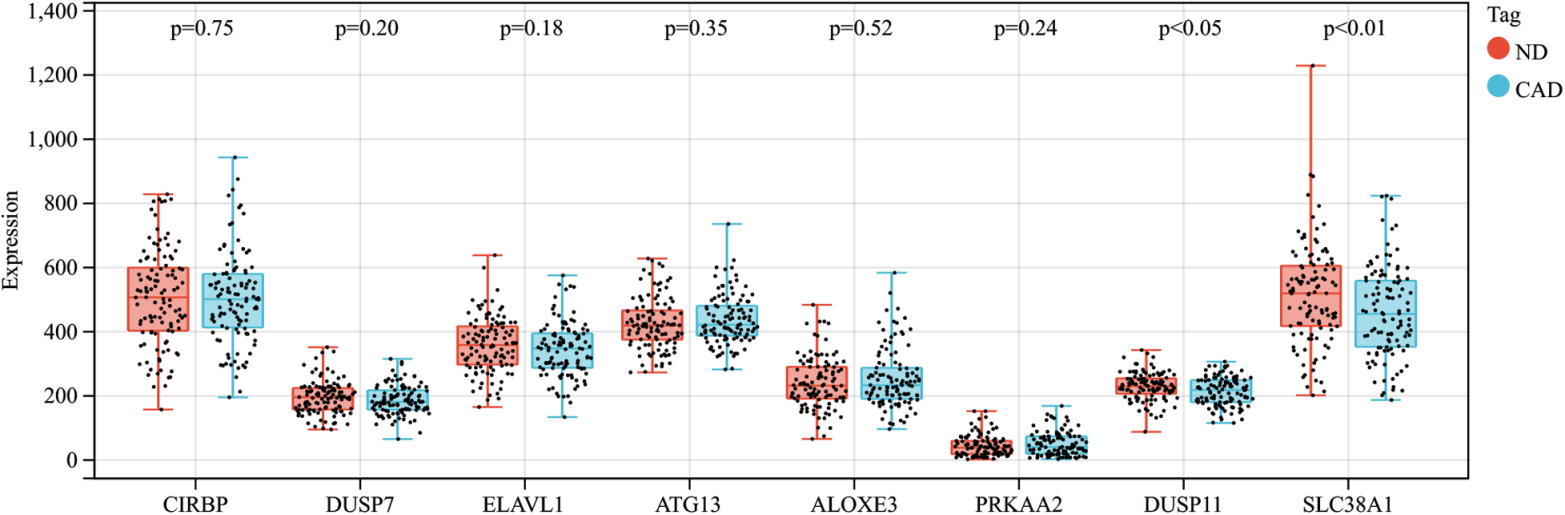
Expression patterns of central genes in CAD vs. Control groups in CAD experimental samples.

In this research, clustering was applied to both samples and genes (as shown in Figures 3C, D, respectively) to discern patterns of similarity in their expression, which helps in understanding gene relationships and the variance in expression across different samples. The correlation between gene modules and clinical traits was explored (Figure 3E), integrating central genes from limma analysis, genes implicated in iron metabolism identified by WGCNA, and other related genes, pinpointed 8 overlapping genes (CIRBP, USP7, ELAVL1, ATG13, ALOXE3, PRKAA2, USP11, SLC38A1), highlighted in Figure 4A. Among these, USP11 and SLC38A1 were found to have statistically significant differential expression. The outcomes of these analyses were visually represented through violin plots in Figure 5.

### 3.2 Enriched biological processes and pathways associated with central genes

Enrichment analysis was performed to gain insights into the potential biological functions of these genes. GO analysis revealed that 6 out of the 8 genes were associated with various biological processes, such as cytoplasmic stress granule, cytoplasmic ribonucleoprotein granule, ribonucleoprotein granule, regulation of cellular catabolic process, regulation of catabolic process, stress granule assembly, cellular catabolic process, catabolic process, mRNA stabilization, and negative regulation of cellular catabolic process (Figure 4B).

### 3.3 Construction and performance of the diagnostic model

**Figure 6:**
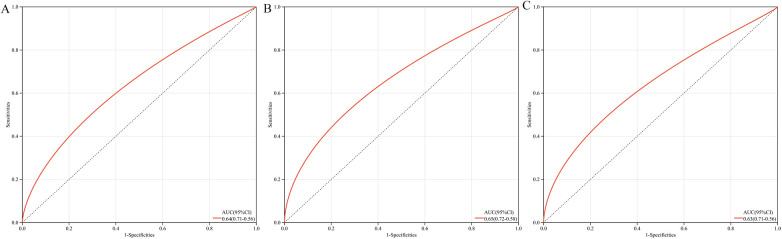
ROC curves and AUC metrics for expression groups. **(A)** For samples from GSE12288. **(B)** For blood samples from GSE60993. **(C)** For blood samples from GSE66360.

The multigene predictive model derived from stepwise logistic regression analysis exhibited strong diagnostic performance, with an AUC value of 0.64 (Figure 6A). Furthermore, this model was subsequently validated in blood samples, showing higher AUC values in GSE60993 and GSE66360, at 0.65 and 0.63 respectively (Figures 6B, C). These results suggest that the model has potential clinical application prospects in diagnosing patients with coronary heart disease.

### 3.4 Immune infiltration

**Figure 7:**
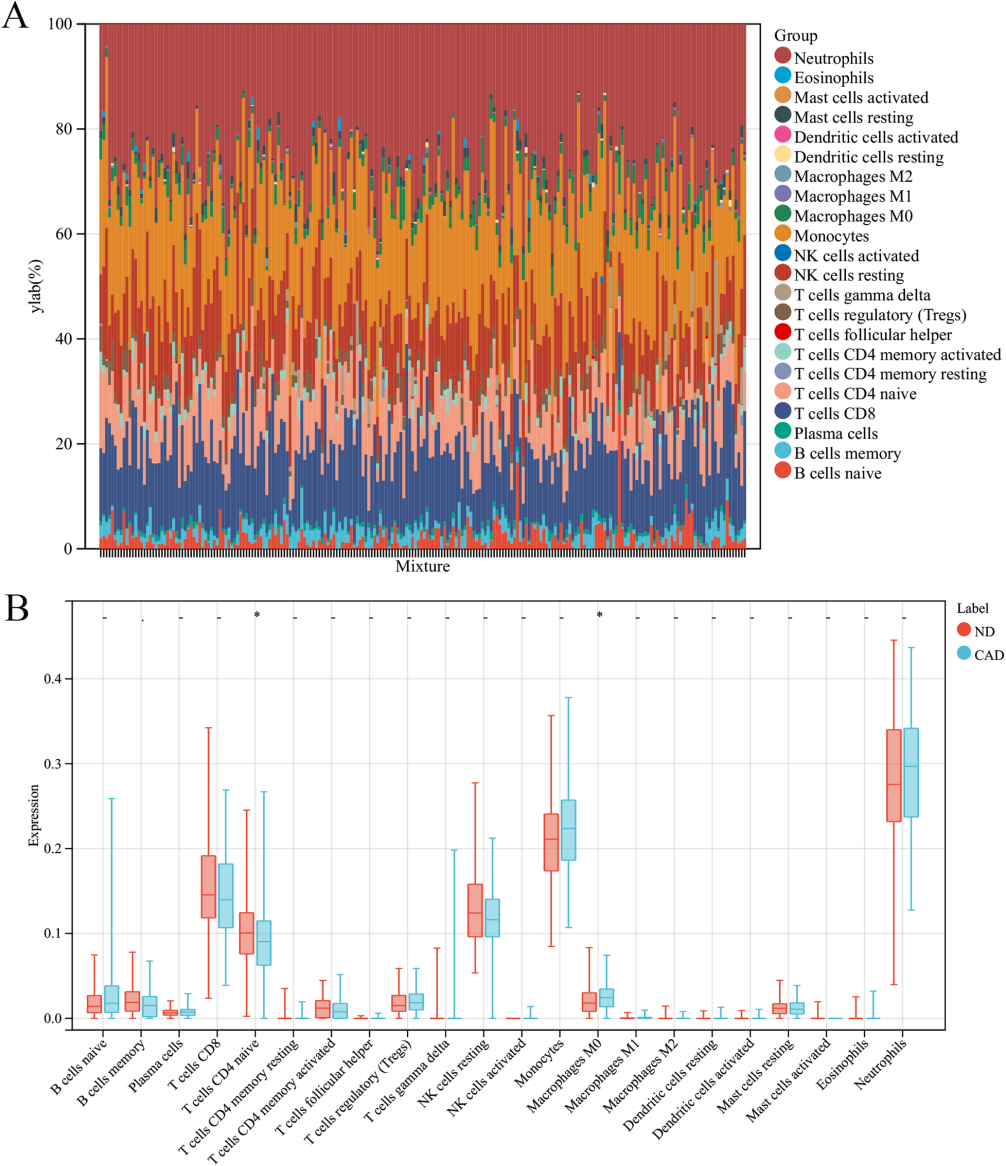
Immune cell composition in CAD vs. ND Samples. **(A)** The proportion of 22 immune cell types in each sample. **(B)** The variation in immune cell infiltration between CAD and ND samples.

In this research, the impact of the microenvironment, which includes immune cells, the extracellular matrix, inflammatory mediators, and growth factors, on the sensitivity to clinical treatments and diagnostic accuracy was examined. Specifically, the study analyzed the composition of 9 different immune cells in 112 samples from patients with coronary heart disease and 110 samples from a healthy control group using the CIBERSORT algorithm. The findings, depicted in a histogram (Figure 7A) and a box plot (Figure 7B), showed that the levels of M0 macrophages were elevated in the coronary heart disease group compared to the healthy controls. Conversely, CD8T cells were found in greater abundance in the healthy group than in the coronary heart disease group.

## 4 Discussion

Coronary heart disease is a cardiovascular disease closely related to oxidative stress and inflammatory reactions, and iron is an important mineral in many physiological and biochemical reactions in the human body. Iron possesses reactive redox capabilities, leading to the accumulation of reactive oxygen species (ROS) and lipid peroxidation (23). Studies have shown that iron metabolism disorder is involved in the pathological progression of coronary heart disease, and the consequences of iron metabolism disorder are related to iron overload-induced programmed cell death (ferroptosis) (24).

The metabolism and regulatory mechanisms of iron in myocardial cells play a crucial role in iron-mediated damage in many cardiovascular diseases (25). The pathways of iron import and export, as well as the intracellular distribution of iron, are essential for heart function and the development of coronary heart disease (26). Iron overload can lead to an increase in free iron within cells, triggering lipid peroxidation and organelle damage, ultimately resulting in cardiac injury (27, 28). Moreover, iron overload also promotes the development of endothelial cells and atherosclerosis.

The accumulation of iron impacts ischemic and hypoxic damage in the heart, leading to abnormal cascades of cell death signals. In addition to the effects of iron, iron metabolism and oxidative stress are closely related to autophagy (15, 29). Proper autophagy can protect myocardial cells by reducing oxidative stress and attenuating myocardial inflammation, but excessive autophagy can lead to myocardial cell death, exacerbating cardiac dysfunction (30). Iron accumulation also induces the release of intracellular iron, further exacerbating myocardial damage (16).

Additionally, ferroptosis, as a novel iron-dependent form of regulatory cell death driven by lipid peroxidation, has been found to play a significant role in ischemic heart disease (4). Studies have shown that myocardial infarction leads to high levels of ROS production in myocardial cells, and ferroptosis inhibitors can significantly reduce the area of myocardial infarction (31). The relationship between iron and coronary artery disease (CAD) involves various aspects such as iron metabolism and regulation, oxidative stress, autophagy, and ferroptosis (32).

We identified eight hub genes (CIRBP, USP7, ELAVL1, ATG13, ALOXE3, PRKAA2, USP11, SLC38A1) that exhibited significant differences between coronary heart disease (CAD) patients and healthy controls. These genes are primarily involved in cellular stress responses, autophagy, and metabolic regulation, suggesting that dysregulation of iron metabolism may promote the development of CAD by affecting these key cellular processes. Specifically, USP11, a member of the ubiquitin-specific protease family, serves as a critical regulator of protein homeostasis through its deubiquitinating activity. This enzyme plays a fundamental role in modulating intracellular iron homeostasis by controlling the stability and turnover of key iron metabolism-related proteins (33, 34). Dysregulation of USP11 expression can significantly disrupt the precise balance of iron metabolism protein degradation pathways, leading to pathological accumulation of iron ions within cellular compartments (35). The resultant excess of free iron ions acts as a potent catalyst for the generation of reactive oxygen species (ROS), triggering oxidative stress cascades that can inflict substantial damage on cardiomyocytes (36). Concurrently, SLC38A1, a sophisticated amino acid transport system, functions as an essential mediator of intracellular amino acid homeostasis. Alterations in SLC38A1 expression patterns can profoundly impact the biosynthesis of iron-regulatory proteins, particularly ferritin, the primary iron storage protein. Compromised ferritin synthesis creates a deleterious cycle where elevated levels of free iron further amplify oxidative stress damage (37, 38). This pathological cascade can subsequently compromise mitochondrial integrity and function, ultimately disrupting the critical energy metabolism pathways in cardiomyocytes, which are essential for maintaining proper cardiac function. CIRBP is a stress response protein that is upregulated under hypoxic and oxidative stress conditions (39). In the context of CAD, abnormal expression of CIRBP may reflect the hypoxic and oxidative stress status faced by myocardial cells, which is closely related to myocardial ischemia caused by coronary artery stenosis (40). USP7 and USP11 are ubiquitin-specific proteases that participate in the regulation of protein homeostasis. Changes in their expression may affect the stability of iron metabolism-related proteins, thereby disrupting the intracellular balance of iron (41–43).ELAVL1 (HuR) is an RNA-binding protein that plays a pivotal role in mRNA stability and translational regulation. Its abnormal expression may influence the expression of genes related to iron metabolism, consequently affecting iron homeostasis (44, 45). ATG13 is a key regulatory factor in the autophagy process, and changes in its expression suggest alterations in autophagy activity, which may affect the cell's ability to respond to iron overload (46). ALOXE3 is involved in lipid metabolism, and its abnormal expression may be associated with lipid peroxidation and iron-dependent cell death (ferroptosis) (47). PRKAA2 (the *α*2 subunit of AMPK) is an important regulator of cellular energy metabolism (48). In CAD, changes in PRKAA2 expression may reflect metabolic dysregulation in myocardial cells, linked to mitochondrial dysfunction caused by iron overload (49). SLC38A1 is an amino acid transporter, and alterations in its expression may affect the intracellular balance of amino acids, further influencing the synthesis of ferritin and other iron metabolism-related proteins (50).The functional network of these hub genes reveals that dysregulation of iron metabolism may participate in the pathological processes of CAD through multiple pathways, including oxidative stress, energy metabolism dysregulation, protein homeostasis imbalance, and autophagy abnormalities. This provides new insights into the molecular mechanisms underlying coronary heart disease.

Furthermore, in our study, we found that macrophages play a crucial role in naive CD4+ T cells in healthy coronary artery disease. Macrophages play a significant role in coronary artery disease (CAD). CAD is a coronary artery vascular disease caused by atherosclerosis and is a major cause of cardiovascular diseases (51, 52). During the development of CAD, macrophages participate in the formation and stability regulation of atherosclerotic plaques (53).

The main roles of macrophages in atherosclerotic plaques include promoting inflammation, regulating plaque stability, and participating in the regression process of plaques (54, 55). They form foam cells by ingesting and accumulating lipoproteins within the vascular wall, promoting lipid deposition and continuous growth of plaques (56, 57). Additionally, macrophages participate in maintaining local inflammatory responses by secreting pro-inflammatory cytokines, chemokines, and generating reactive oxygen and nitrogen species, further promoting plaque development (58, 59). In the regression process of CAD, the transformation of macrophages into anti-inflammatory M2 macrophages may help reduce plaque inflammation, promote plaque regression and stability (60, 61).

CD4+ T cells play a crucial role in adaptive immune responses. Studies have shown that different subgroups of CD4+ T cells are associated with atherosclerosis and coronary artery disease in the atherosclerosis process. Specifically, naive CD4+ T cell subgroups are correlated with atherosclerosis and coronary artery disease (62, 63). Naive CD4+ T cells play a role in the development of atherosclerosis (64). These cells may be activated during the development of atherosclerosis and participate in inflammatory responses (65). Therefore, the number and activity of naive CD4+ T cells may be related to the development of atherosclerosis and coronary artery disease (66). Additionally, analyzing CD4+ T cell subgroups using multi-parameter/multi-color flow cytometry and their correlation with atherosclerosis has been studied (67, 68). The results suggest a correlation between naive CD4+ T cells and atherosclerosis, further supporting the potential role of naive CD4+ T cells in atherosclerosis (69).

In conclusion, the immune system plays a critical role. Immune cells and inflammatory mediators participate in processes such as plaque formation, plaque instability, and thrombus formation, promoting the development and worsening of CAD. The activation and regulation of the immune system have a significant impact on the pathophysiological processes of CAD, making immune modulation a crucial strategy for the treatment and prevention of CAD.

Although this study successfully established a robust diagnostic tool through a multi-gene prediction model (with AUC values ranging from 0.63 to 0.65) and validated it across multiple independent datasets (GSE60993 and GSE66360), certain limitations remain. The diagnostic accuracy of the model has room for improvement, which may somewhat restrict its clinical utility. Future large-scale prospective cohort studies are needed to further validate and optimize the diagnostic performance of this model, with the aim of developing a more precise diagnostic tool for coronary heart disease.

## 5 Conclusion

This study successfully identified iron metabolism genes associated with coronary heart disease by analyzing peripheral blood gene expression data and constructed a logistic regression model with good diagnostic performance. The results indicate that iron metabolism plays a significant role in the pathogenesis of coronary heart disease. Additionally, immune cell type analysis revealed differences between coronary heart disease patients and normal controls. These findings provide important reference for further research on the diagnosis and treatment of coronary heart disease, and offer new research directions for the role of iron metabolism in coronary heart disease.

## Data Availability

The original contributions presented in the study are included in the article/Supplementary Material, further inquiries can be directed to the corresponding author.
